# A chromosome-level haplotype-resolved genome assembly of oriental tobacco budworm (*Helicoverpa assulta)*

**DOI:** 10.1038/s41597-024-03264-6

**Published:** 2024-05-06

**Authors:** Yalong Xu, Chen Wang, Zefeng Li, Xueao Zheng, Zhengzhong Kang, Peng Lu, Jianfeng Zhang, Peijian Cao, Qiansi Chen, Xiaoguang Liu

**Affiliations:** 1 China Tobacco Gene Research Center, Zhengzhou Tobacco Research Institute of CNTC, Zhengzhou, 450001 China; 2 Beijing Life Science Academy (BLSA), Beijing, 102209 China; 3 Institution Henan International Laboratory for Green Pest Control, Henan Engineering Laboratory of Pest Biological Control, College of Plant Protection, Henan Agricultural University, Zhengzhou, 450000 China

**Keywords:** Comparative genomics, Genome informatics, Sequence annotation

## Abstract

Oriental tobacco budworm (*Helicoverpa assulta*) and cotton bollworm (*Helicoverpa armigera*) are two closely related species within the genus Helicoverpa. They have similar appearances and consistent damage patterns, often leading to confusion. However, the cotton bollworm is a typical polyphagous insect, while the oriental tobacco budworm belongs to the oligophagous insects. In this study, we used Nanopore, PacBio, and Illumina platforms to sequence the genome of *H. assulta* and used Hifiasm to create a haplotype-resolved draft genome. The Hi-C technique helped anchor 33 primary contigs to 32 chromosomes, including two sex chromosomes, Z and W. The final primary haploid genome assembly was approximately 415.19 Mb in length. BUSCO analysis revealed a high degree of completeness, with 99.0% gene coverage in this genome assembly. The repeat sequences constituted 38.39% of the genome assembly, and we annotated 17093 protein-coding genes. The high-quality genome assembly of the oriental tobacco budworm serves as a valuable genetic resource that enhances our comprehension of how they select hosts in a complex odour environment. It will also aid in developing an effective control policy.

## Background & Summary

The oriental tobacco budworm *Helicoverpa assulta* (Guenée) and cotton bollworm *H. armigera* (Hübner), commonly known as two sibling species, belong to the order Lepidoptera and the family Noctuidae. They are widely distributed across Africa, Oceania, and Southeast Asia^1^, with both species playing significant roles as pests in agricultural systems. Moreover, they are commonly used as research materials in the field of entomology, boasting a substantial foundation of scientific studies. Morphologically, the two species are nearly indistinguishable at all stages, including the egg, larva, and pupal stages, and only identifiable during the adult stage by certain characteristics^2,3^. Physiologically, they have the same major sex pheromone components of (Z)-9-hexadecenal and (Z)-11-hexadecenal^4^. Despite sharing some characteristics, they display marked variations in host range, resistance to pesticides, ratios of pheromone components, and reproductive capacity. The cotton bollworm is a typical polyphagous insect, able to feed on over 180 plant species, including cotton, maize, soy, wheat, and rice^5^.

Meanwhile, the oriental tobacco budworm primarily infests plants from the Solanaceae family, such as tobacco, tomato, and peppers^6,7^. A noteworthy phenomenon is observed in the relationship between cotton bollworm and oriental tobacco budworm, where despite being distinct species, they exhibit significant genetic similarity, enabling them to interbreed and generate diverse progeny. Specifically, the successful crossing of female *H. assulta* with male *H. armigera* resulted in viable and fertile F1 hybrids. Conversely, the reverse cross of female *H. armigera* with male *H. assulta* produced F1 hybrids, which included fertile males and abnormal individuals but lacked fertile females^7^. Additionally, both species can successfully consume spicy pepper fruits; however, research findings revealed that *H. assulta* demonstrates a higher tolerance to capsaicin derived from *Capsicum annuum* compared to *H. armigera*^6^. Therefore, *H. assulta* is an exemplary model for investigating evolutionary patterns in insect feeding habits and elucidating the underlying mechanisms governing interactions with host plants.

This study presents a high-quality haplotype-resolved genome assembly of *H. assulta* at the chromosome level, achieved through the use of PacBio long reads, nanopore ultra-long reads, and high-throughput chromosome conformation capture (Hi-C) data. Utilizing Hifiasm^8^, we created three haplotype-resolved draft genomes: primary, paternal, and maternal, their genome sizes were 441.6 MB, 395.38 MB, and 404.67 MB, respectively. Following the correction of sequence errors and removal of haplotigs, the primary genome now stands at 415.19 Mb in size, with a contig N50 length of 13.99 Mb. Notably, all 33 primary contigs were successfully anchored onto 32 chromosomes, encompassing both Z and W sex chromosomes.

Furthermore, the genome assembly exhibited a high degree of completeness, as evidenced by the BUSCO analysis, which revealed 99.0% gene coverage. Repeat sequences constituted 38.39% of the genome assembly. A total of 17,093 protein-coding genes were identified, with 16,889 being functionally annotated. Transcriptome analysis indicated that 14,681 genes were expressed in at least one sample.

## Methods

### Sample collection

The larvae of *H. assulta* were collected from tobacco fields in the Xu Chang campus of Henan Agricultural University (113.80° E, 34.13° N) and reared continuously for more than ten generations in the laboratory. The insects were reared on an artificial diet under controlled conditions at 26 ± 1 °C, with a 14:10 (L:D) photoperiod cycle and 85% ± 5% relative humidity. Pupae and newly molted adults were selected for sequencing, and the adult insects that were used for sequencing had their wings removed before the process.

### Genome sequencing and size estimation

The genomic DNA for PacBio HiFi sequencing was extracted from a newly molted female adult using the QIAamp DNA Mini Kit (QIAGEN). The DNA’s integrity was assessed using the Agilent 4200 Bioanalyzer (Agilent Technologies, Palo Alto, California). Subsequently, 15 μg of genomic DNA was sheared using g-Tubes (Covaris) and concentrated with AMPure PB magnetic beads. Each SMRT bell library was prepared using the Pacific Biosciences SMRTbell express template prep kit 2.0. The constructed libraries underwent size selection on a BluePippin™ system for molecules ≥ 15Kb, followed by primer annealing and the binding of SMRT bell templates to polymerases using the DNA/Polymerase Binding Kit. Sequencing was performed on the Pacific Bioscience Sequel II platform for 30 hours at the Annoroad Gene Technology company. Finally, a total of 1,933,848 high-quality HiFi reads were generated with a combined length of 38,425,265,244 bp; the detailed information about HiFi reads is listed in Table 1.

**Table 1:** Summary statistics of the Illumina HiFi reads.

Terms	Statistics
HiFi Reads	1,933,848
HiFi Yield(bp)	38,425,265,244
HiFi Read Length (mean, bp)	19,869
HiFi Read Quality (median)	Q30
HiFi Number of Passes (mean)	8
Below Q20 Reads	383,984
Below Q20 Yield (bp)	8,044,010,604
Below Q20 Read Length (mean, bp)	20,948
Below Q20 Read Quality (median)	Q17

To perform Illumina second-generation DNA sequencing, one newly molted adult female and its parents were collected and rinsed with pre-cooled 0.9% saline to contamination, and frozen with liquid nitrogen. Genomic DNA was extracted from the collected samples using the sodium dodecyl sulfate (SDS) extraction method. After testing the DNA quality and integrity, it was randomly sheared by a Covaris ultrasonic disruptor. Illumina sequencing pair-end libraries were prepared using the Nextera DNA Flex Library Prep Kit (Illumina, San Diego, CA, USA). Sequencing was performed using the Illumina NovaSeq 6000 platform (Illumina, San Diego, CA, USA). Raw reads were filtered using Fastp^9^ software (version 0.21.0) with the following criteria: removal of reads with adapter contamination, removal of reads with an N proportion greater than 5%, and discarding reads with a low-quality base count of 50% or more, where the quality value is less than or equal to 19 (Table 2).

**Table 2:** Summary statistics of the Illumina genomic DNA short reads.

Samples	Usage	Insertion Size (bp)	Sequence Number	Total base (bp)	Coverage depth
HiFi-sister1	Genome survey	350	271,271,596	40,690,739,400	94.63
HiFi-mather	Trio-partition	350	152,276,580	22,841,487,000	53.12
HiFi-father	Trio-partition	350	154,381,868	23,157,280,200	53.85
HiFi-sister2	Hi-C assembly	350	154,726,330	23,208,949,500	53.97

The Hi-C libraries were constructed using standard protocols as previously described^10^, with one newly molted female used as the input. The Hi-C sequencing library was then amplified by PCR (12–14 cycles) and sequenced on the Illumina HiSeq instrument, generating 154,726,330 paired clean reads with 2 × 150-bp reads.

We collected female pupae specifically for the construction of Oxford Nanopore libraries. The libraries were prepared using the standard protocol for Oxford Nanopore sequencing, specifically the Ultra-Long DNA Sequencing Kit protocol (SQK-ULK001). The purified library was loaded onto primed R9.4 Spot-On Flow Cells and sequenced using a PromethION sequencer (Oxford Nanopore Technologies, Oxford, UK) with 72-hour runs at Novogene Corporation Inc., Tianjin, China. Basecalling of raw fast5 format data was performed using Oxford Nanopore GUPPY^11^ software, removing low-quality reads with a sequencing quality value (Q) less than seven and retaining high-quality pass reads. The quality assessment report was generated using NanoPlot^12^ v1.38.1. Finally, 254,496 Oxford Nanopore raw reads were generated with a combined length of 25,843,417,397 bp, and the detailed information is listed in Table 3.

**Table 3:** Summary statistics of the Oxford Nanopore raw reads.

Base	Number	N50	Length (mean)	Quality (mean)	Quality > Q7	Quality > Q10
25,843,417,397.0	254,496.0	100,000.0	101,547.0	13.5	95.6%	86.8%

**Fig. 1:**
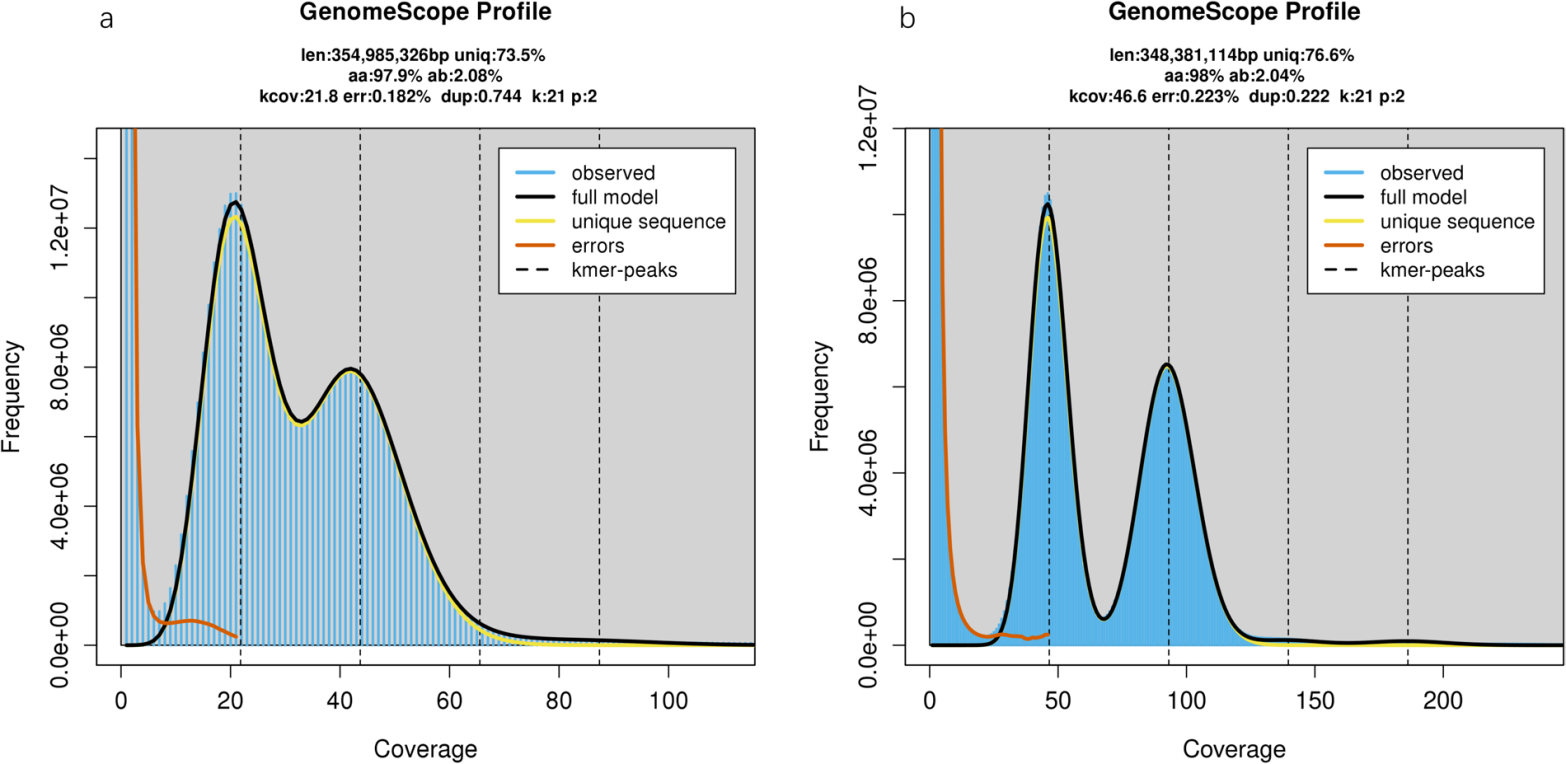
K-mer spectra and fitted models for *H. assulta* based on Illumina short-read reads and PacBio HiFi reads using a 21-mer count histogram. (**a**) K-mer spectra and fitted models for *H. assulta* based on Illumina short-read reads. (**b**) K-mer spectra and fitted models for *H. assulta* based on PacBio HiFi reads.

Genomic characteristics, such as genome size, repeat content, and heterozygous rate, were estimated based on K-mer frequencies. Utilizing K-mer analysis (K = 21) of Illumina short reads and PacBio HiFi long reads with Jellyfish^13^ v2.3.0, we estimated the overall genome size of *H. assulta* to be approximately 350 Mb using genescope2.0^14^. For the Illumina short-read reads, the genome size was estimated to be 354.98 Mb, with a heterozygosity rate of 2.08%; For the PacBio HiFi reads, the genome size was estimated to be 348.38 Mb, with a heterozygosity rate of 2.04% (Fig. 1).

### Genome assembly

The chromosome-level haplotype-resolved genome assembly with trio binning was achieved using Hifiasm^8^ v0.19.5 software; this involved incorporating Illumina short paired-end reads from the parents, Illumina Hi-C paired-end reads, ultra-long ONT reads, and Pacbio HiFi reads. The primary contigs and two other haplotypes (paternal and maternal) contigs assembled by Hifiasm were further refined using Nextpolish2^15^ v0.1.0 software. This refinement process involved the use of PacBio long HIFI reads and Illumina short reads, resulting in the production of three draft genome assemblies.

Certain regions in a genome with high genetic diversity result in separate primary contigs for each haplotype instead of a single contig with an associated haplotig^16^. Whether you are working on the haploid or phased-diploid assembly, this can be an issue for downstream analysis. Hifiasm^8^ is a powerful assembler that can generate high-quality chromosome-level assemblies. Compared to other assemblers, it produces longer contigs and can resolve more segmental duplications. By using Hifiasm, we created three haplotype-resolved draft genomes: primary, paternal, and maternal, their genome sizes were 441.6 MB, 395.38 MB, and 404.67 MB, respectively (Table 4). Although Hifiasm can eliminate most duplications between haplotigs, it may incorrectly identify or fail to distinguish some heterozygous sequences. To address this issue, we used the Purge Haplotigs^17^ v1.1.2 software with long HiFi reads to remove haplotigs remaining in the three draft assembled genomes.

**Table 4:** Summary statistics of three draft Hifiasm assemblies for *H. assulta*.

Statics	Paternal (Hap1)	Maternal (Hap2)	Primary
Number of contigs	109	90	111
Percent GC (%)	37.22	37.38	37.46
Contig N50 (bp)	13,230,218	13,847,332	13,795,863
Average length (bp)	3,627,347.21	4,496,358.08	3,978,652.33
Total assembled bases	395,380,846	404,672,227	441,630,409

Assembly completeness was estimated by BUSCO^18^ v5.4.7 analysis and Illumina short reads mapping; the lineage dataset used in BUSCO is insecta_odb10, and bowtie2^19^ v2.5.1 software was used to align the purged genome assembly. The analysis identified 99.0% (single-copied genes: 98.7%, duplicated genes: 0.3%), 0.5%, and 0.5% of the 1,367 predicted genes in this genome as complete, fragmented, and missing sequences, respectively. These results suggested that the assembled genome is highly complete.

### Genome scaffolding

**Fig. 2:**
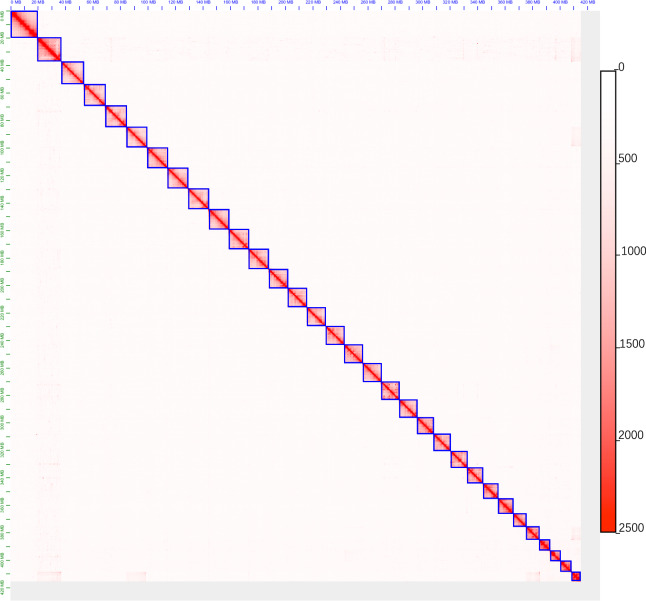
Heat map of Hi-C assembly of *H. assulta*. The scale bar represents the interaction frequency of Hi-C links.

These high-quality Hi-C sequencing clean reads were mapped to the trimmed draft genome using BWA 0.7.17^20^ and filtered for unmapped and multiple mapped reads using Samtools v1.16^21^. The unique, high-quality paired-end reads mapped close to the restriction sites were retained for downstream analysis in the juicer^22^ v1.6 and 3d-dna^23^ v180922 pipeline. Juicebox^23^ was used to cluster the contigs into groups, and the order of the contigs was confirmed based on the strength of interactions between read pairs. During the process of grouping contigs based on Hi-C data, we observed that 33 contigs were grouped into 32 clusters (Fig. 2), with only one cluster (Chr16) containing two contigs. To ensure the accuracy of the connection between these two contigs, we used paired-end information from the sequencing data, if there are telomere repeat sequences present, confirm that they are located at the ends of the sequence. After correcting sequence errors and removing haplotigs, the final genome stands at 415.19 Mb, with an average length of 12.58 Mb after scaffolding (Table 5).

**Table 5:** Summary statistics of the final *H. assulta* genome assemble.

Terms	Statistics
Median length (bp)	13,331,090
Average length (bp)	12,581,573.48
Total assembled bases (bp)	415,191,925
Number of chromosomes	32
Total length of chromosomes (bp)	415,191,925
Percent GC (%)	37.30

### RNA sequencing and analysis

**Fig. 3:**
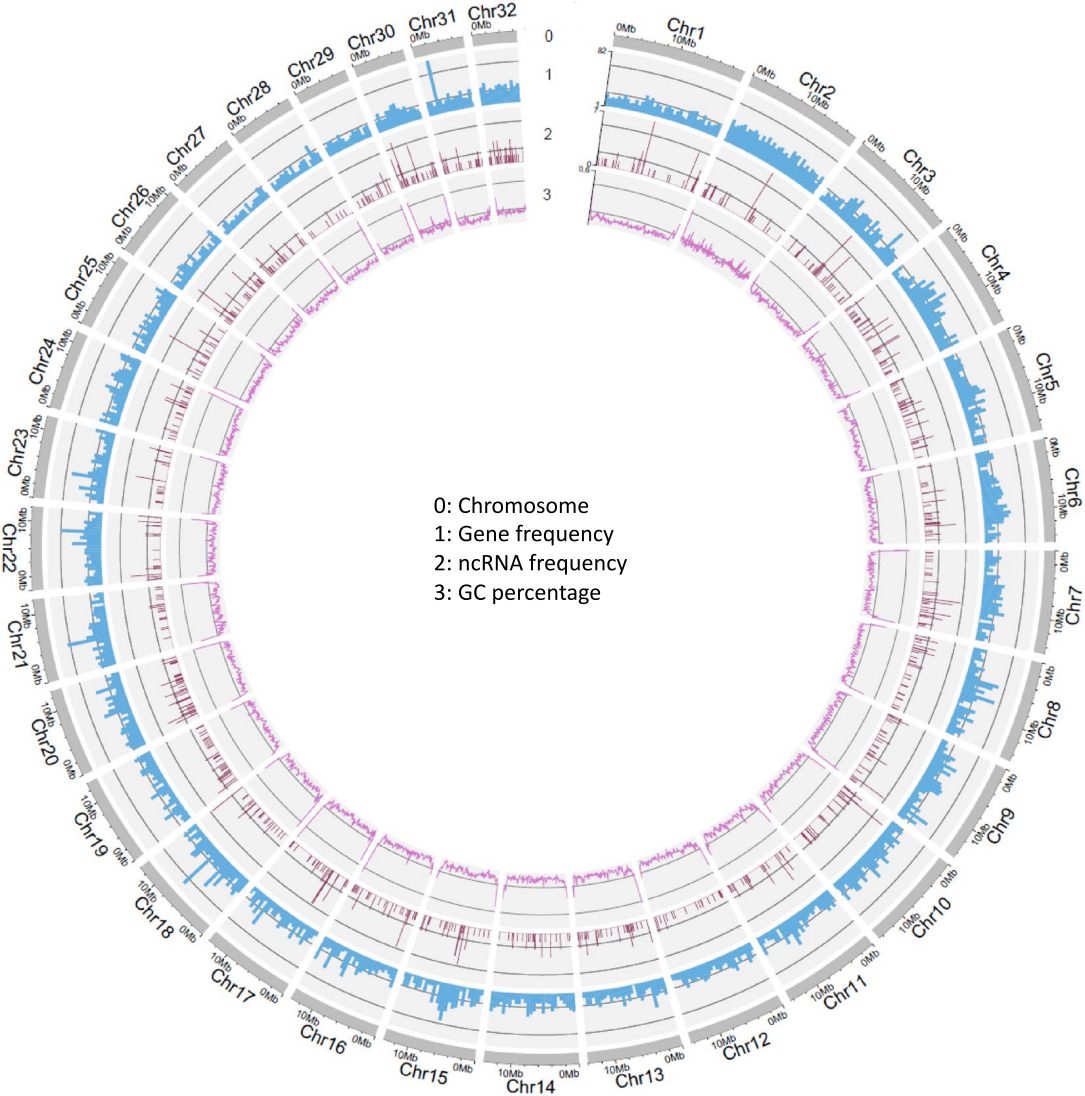
The alignment rate of the RNA-seq data. The RNA-seq data with a dark red colour (**a**) comes from this genome sequencing project; the data with a dark grey colour (**b**) was downloaded from the NCBI SRA database. The value at the red dash-line is equal to 85.

We collected fourth and fifth instar larvae, female and male pupae, and newly emerged male and female adult moths for transcriptome sequencing and gene expression analysis. Before preparation and sequencing, we removed the midguts of the larvae and the wings of the adults. Subsequently, total RNA was extracted from the aforementioned samples using Trizol reagent (Invitrogen, USA) following the manufacturer’s protocol. Illumina RNA sequencing libraries were prepared by Annoroad Gene Technology Company. We performed RNA sequencing on 18 samples and obtained RNA-seq data with a total length of about 1209 gigabytes (Table 6). The total number of sequences is around 807 million, with an average proportion of bases having a quality greater than Q30 at 93.9% and an average proportion of clean reads at 97.25%. Clean data was obtained by removing adapters, low-quality reads, and high-content unknown sequences. All RNAseq data sequenced in this project have been deposited into the European Nucleotide Archive (ENA) with accession number PRJEB7091153. In addition to our sequencing data, we downloaded 39 transcriptome datasets from the NCBI SRA database which were merged with our dataset. Each sample’s data was aligned to the genome using HISAT2^24^ to assess gene transcription levels. Analysis shows that more than half of the transcriptome samples exhibit a genome alignment rate of over 85%, and the genome alignment rate of the samples in this project (group a) is consistently around 90% (Fig. 3).

**Table 6:** Summary statistics of the Illumine RNA-seq short reads.

Sample	Total Raw Reads	Total Raw Bases (bp)	Total Clean Reads	Clean Reads Rate (%)	Total Raw Bases (bp)	Clean Q30 Bases Rate (%)	Clean GC percent (%)	Sample Information
4L_1	46295682	6.94 G	45042726	97.29	6.76 G	93.89	49.6	4th instar larva
4L_2	42994866	6.45 G	41901426	97.46	6.29 G	93.94	49.84	4th instar larva
4L_3	42282222	6.34 G	41152134	97.33	6.17 G	93.66	49.89	4th instar larva
5L_1	47690030	7.15 G	46188932	96.85	6.93 G	94	51.47	5th instar larva
5L_2	47963800	7.19 G	46622096	97.2	6.99 G	94.59	51.54	5th instar larva
5L_3	48859300	7.33 G	47406188	97.03	7.11 G	94.08	51.17	5th instar larva
female_1	41383760	6.21 G	40581714	98.06	6.09 G	93.32	45.93	female adult
female_2	43216260	6.48 G	42003220	97.19	6.3 G	93.32	47.94	female adult
female_3	46920670	7.04 G	45500488	96.97	6.83 G	93.59	46.94	female adult
male_1	39857922	5.98 G	38868690	97.52	5.83 G	93.76	48	male adult
male_2	42061634	6.31 G	41137486	97.8	6.17 G	93.98	46.91	male adult
male_3	39731146	5.96 G	38741222	97.51	5.81 G	94.85	47	male adult
pupa_M_1	45722346	6.86 G	44145206	96.55	6.62 G	94	50.79	male pupa
pupa_M_2	46218566	6.93 G	44977156	97.31	6.75 G	93.82	51.47	male pupa
pupa_M_3	45124066	6.77 G	44014818	97.54	6.6 Gb	93.9	50.45	male pupa
pupa_F_1	46709998	7.01 G	45379916	97.15	6.81 G	94.13	50.65	female pupa
pupa_F_2	47051248	7.06 G	45889672	97.53	6.88 G	93.68	50.35	female pupa
pupa_F_3	45993886	6.9 Gb	44244766	96.2	6.64 G	93.62	50.81	female pupa

### Genome annotation

We first aligned the RNA-seq data mentioned above to the final genome using HISAT2^24^ v2.2.1 and then predicted the transcripts with StringTie^25^ v2.2.1. TACO^26^ v0.73 was employed to merge the transcripts, retaining the high-quality ones. Next, we utilized TransDecoder v5.7.1 (https://github.com/TransDecoder/TransDecoder) to predict the protein-coding sequence. We initially built a de novo transposable elements (TE) library using the EDTA^27^ v2.1.0 pipeline for repeat sequence annotation with the CDS file obtained from the TransDecoder results. Subsequently, we masked repeat sequences across the *H. assulta* genome using RepeatMasker^28^ v4.1.2 against the de novo species-specific TE library generated by EDTA and the insect data from Dfam^29^ 3.6.

Following the masking of these TE sequences, we integrated ab initio prediction, homology searching, and transcriptome-based approaches to predict protein-coding genes using the BRAKER3^30^ pipeline with the parameters “--bam RNAseqs.bam --prot_seq = Arthropoda.10.pep.fa --min_contig = 10000 --addUTR = on --gff3 --threads = 48”. The annotated proteins of all arthropods were downloaded from OrthoDB^31^ v10, and RNA-Seq alignment bam files were generated by HISAT2. We used eggNOG-mapper^32^ v2.1.12 for functional annotation. Additionally, we searched the Uniprot^33^ database using Blastp^34^ v2.14.1 + and the Pfam^35^ and KOfam^36^ databases using HMMER^37^ v3.4.

**Fig. 4:**
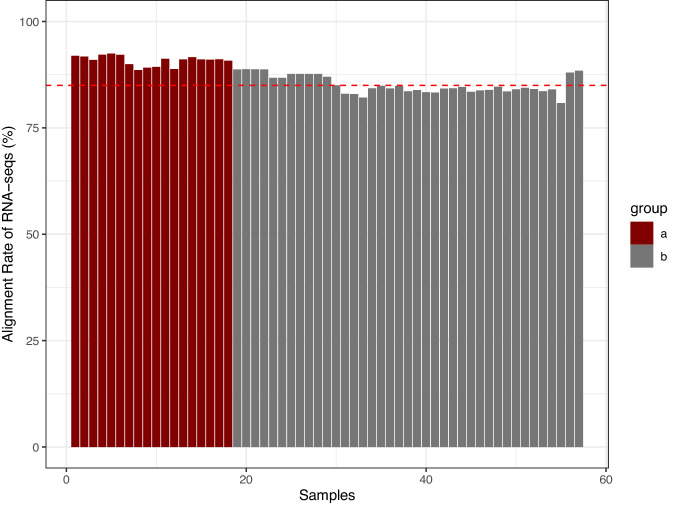
Characterization of the *H. assulta* genome. Circos plot of chromosome level genome assembly (~415.19 Mb) and the distribution of GC content, gene frequency, and ncRNA frequency on 32 chromosomes.

In the *H. assulta* genome, a total of 159.38 Mb sequences (38.39%) were identified as repetitive elements, as shown in Table 7. A total of 17,093 protein-coding genes were identified, with 16,889 being functionally annotated and expression analysis indicates that 14,681 genes were expressed in at least one sample (Table 8). In addition, we identified 86 rRNAs and 62 tRNAs. The Circos plot of the functional element we identified is shown in Fig. 4. All annotation files have been deposited into figshare.com^38^.

**Table 7:** Repeats elements statistics of the *H. assulta* genome.

Types	Number	Length (bp)	Percentage (%)
Retroelements	149145	50854396	12.25
SINEs	907	69220	0.02
LINEs	20114	10275506	2.47
LTR elements	128124	40509670	9.76
DNA transposons	712024	89626531	21.59
Rolling-circles	8545	6500.52	0.16
Unclassified	100938	18902911	4.55
Total interspersed repeats	NA	159383838	38.39
Small RNA	720	103079	0.02
Satellites	2	105	0
Simple repeats	65603	3071613	0.74
Low complexity	8896	405688	0.1

**Table 8:** The statistical data on chromosome length, total gene count, and number of expressed genes.

Chromosome	Length (bp)	Total Gene Number	Expressed Gene Number
Chr1 (Z)	19812103	668	579
Chr2 (W)	17568800	1047	514
Chr3	16235540	743	679
Chr4	15571065	756	686
Chr5	15450104	625	543
Chr6	15058675	678	631
Chr7	15011965	593	513
Chr8	14987496	637	552
Chr9	14842725	633	575
Chr10	14715802	669	598
Chr11	14501914	557	495
Chr12	14490050	513	460
Chr13	14013935	504	439
Chr14	13996836	551	497
Chr15	13699072	574	530
Chr16	13472289	525	465
Chr17	13331090	489	409
Chr18	13245517	649	595
Chr19	13179736	458	376
Chr20	13019475	564	503
Chr21	12259601	526	475
Chr22	12120753	568	512
Chr23	11990349	493	424
Chr24	11668445	508	424
Chr25	11100899	423	354
Chr26	10685235	329	272
Chr27	9723748	242	215
Chr28	9523101	269	241
Chr29	7999195	258	217
Chr30	7688887	355	302
Chr31	7662109	356	311
Chr32	6565914	333	295
Total	415191925	17093	14681

Note: The expression matrix^59^ has been deposited into figshare.com.

### Sex chromosomes analysis

**Fig. 5:**
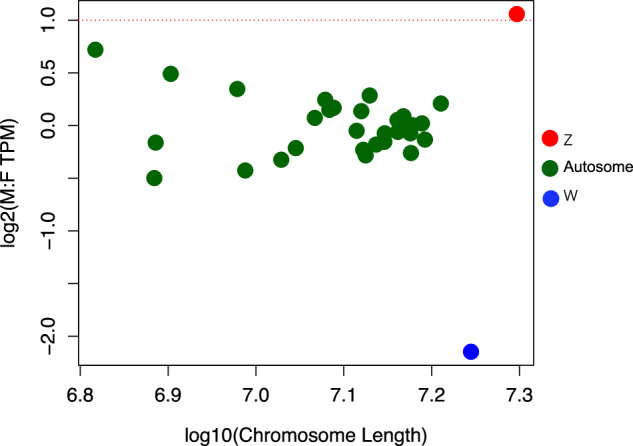
The coverage ratios of male/female for each chromosome. Each point represents a single chromosome. The dotted red line shows the expectation for the Z chromosome.

To identify the sex chromosomes (Z and W chromosomes) in *H. assulta*, we resequenced one female pupa and one male pupa using Illumina HiSeq platforms to obtain an approximate 50 × coverage. In males, the normalized coverage levels of sequence reads from the Z chromosome should be twice that of females. On the other hand, ideally, males do not have any DNA contribution from the W chromosome, while the autosomes should have equal coverage between males and females. Therefore, a difference in sequencing coverage ratio is expected for both Z and W chromosomes between sexes but not for autosomes. This difference can be used to identify sex-linked chromosomes. Using salmon^39^, we computed the normalized coverage levels of chromosomes by mapping the resequencing reads to the final *H. assulta* genome with default parameters. To analyze and visualize the log2 of the male: female (M: F) coverage ratio, we used the R package changepoint v2.2.4 (https://github.com/rkillick/changepoint/). Remarkably, among all the chromosomes, it was observed that the sequencing depth of the longest chromosome (Chr1) is twice as high in males compared to females, leading to the conclusion that Chr1 is the Z chromosome (Fig. 5). Ideally, the length of the W chromosome should be similar to that of the Z chromosome and exhibit shallow sequencing depth in males. Only the second-longest chromosome (Chr2) meets both criteria, thus leading to the conclusion that Chr2 is the W chromosome.

### Synteny analysis

**Fig. 6:**
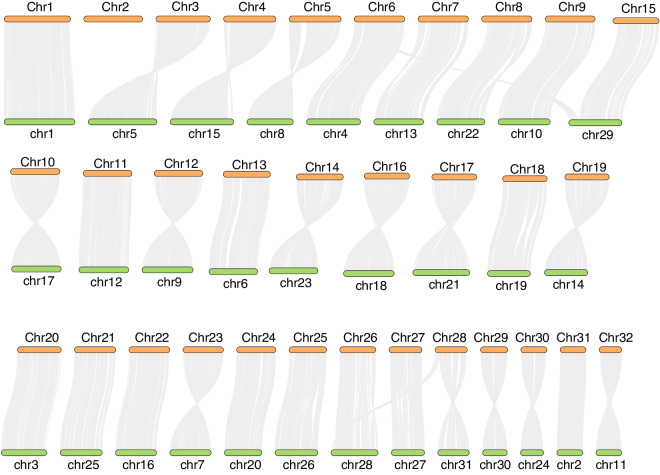
Chromosomal synteny plot of *H. assulta* and *H. armigera* genomes. The dark yellow strip at the top represents the chromosomes of the *H. assulta*, while the light green strip at the bottom represents the chromosomes of the *H. armigera*.

To compare the genomic arrangement of *H. assulta* with its closely related species, cotton bollworms (*H. armigera*), we used annotated protein sequences anchored on chromosomes to perform synteny analysis through MCScanX^40^ with default parameters. From the NCBI genome database, we obtained the reference genome HaSCD2 data (accession number: GCF_023701775.1) of cotton bollworms. The analysis showed that most of the chromosomes of the two moths exhibited good collinearity, with only a few chromosome fragments undergoing fission and fusion events. For example, although most of Chr6 of *H. assulta* was syntenic to Chr4 of *H. armigera*, a small part was syntenic to Chr29. We visualized the results using Tbtools^41^. Due to the absence of the W chromosome in the cotton bollworm reference genome, we did not observe any collinearity between the W chromosome of the *H. assulta* and any chromosome in the cotton bollworm genome (Fig. 6).

### Phylogenetic reconstruction

To establish the evolutionary relationship between the tobacco budworm and other closely related species, we retrieved protein sequences of six species belonging to the Noctuidae family and one Coleopteran insect (*T.castaneum*) from the NCBI genome database and only the longest transcript for each gene was taken into consideration. OrthoFinder^42^ v2.5.4, with DIAMOND^43^ v2.1.8, was used to identify orthologs and homologs. OrthoFinder successfully assigned 125918 genes (96.9%) to 14619 orthogroups. At least 50% of all genes belonged to orthogroups with eight or more genes (G50 was 8) and were contained in the largest 5245 orthogroups (O50 was 5245). There were 6498 orthogroups with all species present, and 2822 of these consisted entirely of single-copy genes.

For the phylogenetic analysis, we constructed a maximum likelihood phylogenetic species tree using the STAG method in the OrthoFinder^42^ program, rooted in STRIDE^44^. Multiple sequence alignments of single-copy gene families were performed using MAFFT^45^ v7.520 with the “-auto” parameter, and the alignment results were trimmed using trimAL^46^ v1.4.rev15 with the “-automated1” setting. The alignments of all single-copy orthologs were concatenated to form a supergene.

We then utilized the mcmctree from the PAML^47^ package to estimate the divergence time of the species in the tree. Divergence information obtained from the TimeTree^48^ database (*S. frugiperda* vs *S. litura* 16.9–19.1 MYA, *N. ni* 70–80, and *T. castaneum* 195–361.6 MYA) was combined with mcmctree to constrain the divergence estimate. Subsequently, we visualized the time tree using the Figtree software (https://github.com/rambaut/figtree). The divergence time distance between *H. assulta* and *H. armigera* was estimated to be around 6.2 million years.

**Fig. 7:**
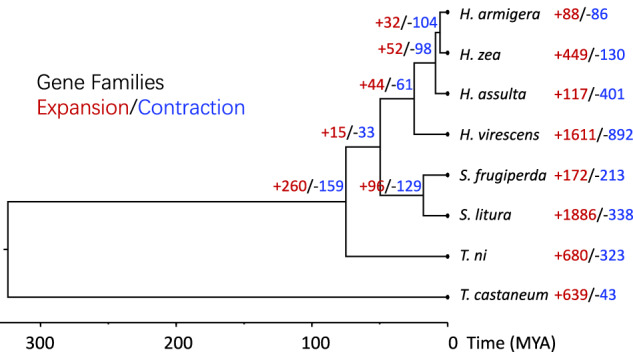
Analysis of the evolution of phylogenetics and gene families in *H. assulta* and seven other species. Node values show the number of gene families that expanded (+) or contracted (−). *T. castaneum* (Coleoptera) was used as an outgroup. The scale at the bottom of the figure indicates divergence time.

To analyze the expansion and contraction of gene families, we utilized the matrix tables of gene family orthologs obtained from OrthoFinder results. We applied these tables as inputs in CAFE^49^ v5.0.0 and set a cut-off p-value of <0.05, allowing us to examine each gene family’s expansion and contraction (Fig. 7).

## Data Records

The Nanopore, Hi-C, and Illumina sequencing data used for the genome assembly and annotation have been submitted to the European Nucleotide Archive (ENA) with accession number PRJEB70911^50^. The final chromosome assembly has been submitted to the National Genomic Data Center (NGDC) under the accession GCA_963856015.1^51^. The *H. armigera* genome was downloaded from the NCBI genome database^52^. All public RNA-seq datasets used in the gene expression analysis were downloaded from the NCBI SRA database, and the corresponding project IDs were PRJEB6594^53^, PRJNA587871^54^, PRJNA590047^55^, PRJNA592822^56^, and PRJNA261645^57^.

## Technical Validation

The chromosome-level primary genome assembly was 415.19 Mb. For quantitative assessment of genome assembly, BUSCO^18^ analysis results showed that 99.0% of BUSCO genes (insecta_odb10) were successfully identified in the genome assembly, suggesting a remarkably complete assembly of the *H. assulta* genome. In addition, the genome alignment rate of HiFi reads is as high as 99.98%. The Hi-C heatmap revealed a well-organized interaction contact pattern along the diagonals within/around the chromosome inversion region, which indirectly confirmed the accuracy of the chromosome assembly.

To verify the completeness of our genome chromosome assembly, we conducted an analysis of telomere repeat sequences on each chromosome based on the genome repeat sequence annotation results. Initially, we analyzed the telomere repeat motif sequences of Lepidoptera insects in TeloBase^58^, and we found that the majority of repeat motifs ranged from 5 to 9 bp in length, and (TTAGG)n/(CCTAA)n is the main motif in telomeres. After that, we identified regions within 15 kb at both ends of the chromosomes in our results where the length of repeat sequences exceeded 1k,and the repeat motif sequence ranged from 5 to 9 bp. Based on our analysis, we found that 21 chromosomes contain the typical telomeric motif (TTAGG)n/(CCTAA)n or a variant of the motif within 15 kb at both ends, while the remaining 11 chromosomes have the typical telomeric motif or a variant of the motif on at least one end (Table 9).

**Table 9:** Information of the telomere repeat sequence motif within 15 kb from both ends of the chromosomes with a length of over 1 kb and closest to the ends.

Chromosome Name	5′-end			3′-end		
	Start	End	Motif	Start	End	Motif
Chr1	1	1556	(ACCTA)n	19807529	19812103	(AGGTT)n
Chr2	1	2315	(ACCTA)n			
Chr3	391	3362	(AACCT)n	16229944	16235540	(AGGTT)n
Chr4	1	2815	(CTAAC)n	15565169	15571065	(GGTTA)n
Chr5	1	1728	(ACCTA)n	15445377	15450104	(AGGTT)n
Chr6	1	6467	(CCTAA)n	15051787	15058675	(GGTTA)n
Chr7	1	1910	(AACCT)n	15010239	15011965	(AGGTT)n
Chr8	10	4542	(CTAAC)n	14980285	14987496	(AGGTT)n
Chr9	1	7401	(CCTAA)n	14839593	14842713	(GGTTA)n
Chr10				14713427	14715802	(GGTTA)n
Chr11	4	2497	(CTAAC)n	14498672	14501252	(AGGTT)n
Chr12	13	7077	(ACCTA)n	14488187	14490050	(GGTTA)n
Chr13				14012745	14013931	(GGTTA)n
Chr14	1	3014	(TAACC)n	13989142	13996833	(AGGTT)n
Chr15	1	2136	(CTAAC)n			
Chr16				13468936	13472287	(GGTTA)n
Chr17	1	3016	(CTAAC)n	13328664	13331090	(AGGTT)n
Chr18	3224	6922	(AACCT)n	13242398	13245029	(GGTTA)n
Chr19	1	1875	(CTAAC)n	13169987	13179736	(GGTTA)n
Chr20	1	5031	(CTAAC)n			
Chr21	1	1613	(AACCT)n	12257237	12259601	(GGTTA)n
Chr22	1	5507	(CTAAC)n	12117784	12120753	(GGTTA)n
Chr23	1	4916	(TAACC)n	11987869	11990349	(AGGTT)n
Chr24	723	3756	(CTAAC)n			
Chr25	5	1627	(CCTAA)n	11098879	11100899	(GGTTA)n
Chr26	692	2487	(CTAAC)n	10681706	10685235	(AGGTT)n
Chr27	1	7612	(TAACC)n	9721154	9723742	(AGGTT)n
Chr28	1	4410	(AACCT)n			
Chr29	1	4385	(CTAAC)n	7994753	7999195	(AGGTT)n
Chr30				7686268	7688887	(AGGTT)n
Chr31				7653763	7662109	(GGTTA)n
Chr32				6562912	6565914	(AGGTT)n

**Fig. 8:**
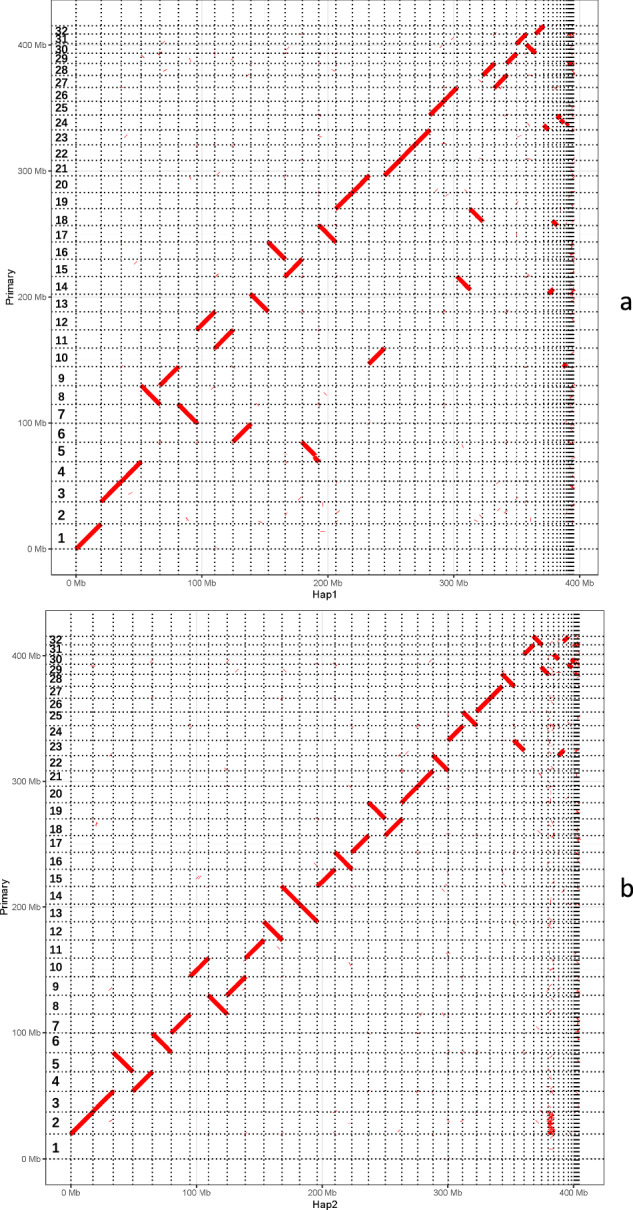
The alignment dot plot of haplotype assemblies Hap1 and Hap2 with the primary reference genome. (**a**) The alignment dot plot of haplotype assemblies Hap1 (paternal haplotype) with the primary reference genome. (**b**) The alignment dot plot of haplotype assemblies Hap2 (maternal haplotype) with the primary reference genome. The two haplotype draft genome sequences^60^ have been deposited into Figshare.com.

In our investigation of sex chromosome determination, we utilized minimap2 to align genome contigs from two haplotypes (paternal and maternal) generated by the Hifiasm program with the primary final genome. The alignment revealed that contigs from the paternal haplotype could be aligned with all chromosomes except Chr2, while those from the maternal haplotype could be aligned with all chromosomes except Chr1 (Fig. 8). It is well-established that the sex determination in tobacco hornworms relies on two sex chromosomes, Z and W, where females possess a Z-W genotype while males have Z-Z. For this study, we employed single-headed female insects as the experimental material for genome sequencing. The analysis above reaffirmed our conclusion that Chr1 is the Z sex chromosome and Chr2 is the W sex chromosome.

## Data Availability

All bioinformatic tools were executed following their respective protocols and manuals. The software version used was described in Methods. Below is detailed parameter information about some bioinformatics tools. **Genome size estimation** jellyfish count -C -m 21 -s 50000000000 -t 32 reads_R*.fq -o reads.jf jellyfish histo -t 32 reads.jf >reads.histo genomescope.R -i reads.histo -o output_dir -k 21 **Genome assembly** hifiasm -o hass --primary -t 48 --h1 hic_read1.fq.gz --h2 hic_read2.fq.gz \ --ul ont.reads.fq.gz hifi_reads.fastq.gz 2 > asm.log yak count -k31 -b37 -t16 -o pat.yak paternal.fq.gz yak count -k31 -b37 -t16 -o mat.yak maternal.fq.gz hifiasm -o hass -t 48 -1 pat.yak -2 mat.yak /dev/null 2 > asm.trio.log **Purge haplotigs** minimap2 -t 48 -ax map-hifi hass.p_ctg.fa hifi_reads.fastq.gz --secondary = no | samtools sort -@ 48 -m 1 G -o hifi_read.aln.bam -T tmp.align purge_haplotigs hist -b hifi_read.aln.bam -g hass.p_ctg.fa -t 48 purge_haplotigs cov -i hifi_read.aln.bam.gencov -l 15 -m 68 -h 140 purge_haplotigs purge -g hass.p_ctg.fa -c coverage_stats.csv -t 48 **Genome sequences correction** yak count -t 48 -k 21 -b 37 -o k21.yak femal.illumina.reads.gz yak count -t 48 -k 31 -b 37 -o k31.yak femal.illumina.reads.gz nextPolish2 -t 48 -o curated.np2.fasta hifi_read.aln.bam curated.fasta k21.yak k32.yak **Hi-C data analysis** juicer.sh -s DpnII -g hass -z curated.np2.fasta -t 60 -p chrom.sizes **Busco analysis** busco -m genome -i genome.fasta -l insecta_odb10 -o busco_out --cpu 45 –offline **HiFi reads mapping** minimap2 -t 48 -ax map-hifi genome.fasta hifi_reads.fastq.gz > hifi_read.aln.sam **Transcript assembling** hisat2 -p 48 -q -x genome.index -1 $j.1.fq.gz -2 $j.2.fq.gz -S $j.sam samtools view -bS -@ 10 -o $j.bam $j.sam samtools sort -@ 10 -o $j.sorted.bam $j.bam stringtie $j.sorted.bam -p 16 -o $j.gtf ls *.gtf > gtf.list taco_run -p 16 gtf.list **Repeat annotation** EDTA.pl --genome genome.fa --cds transcript.cds --sensitive 1 --threads 45 --anno 1 --overwrite 1 --species others --force 1 RepeatMasker -lib repeat.lib -pa 48 -html -xsmall -gff genome.fa > repeatmasker.log **Gene prediction** braker.pl --species = hass I am running a few minutes late; my previous meeting is running over. --genome = genome.fa.mod.MAKER.masked I am running a few minutes late; my previous meeting is running over. --bam rna.aln.bam \ --prot_seq = Arthropoda.10.pep.fa \ --gff3 --threads = 48 --workingdir = braker3_out --min_contig = 10000 --overwrite --addUTR = on **Genome annotation** emapper.py -i pep.fa -o pep.fa --itype proteins --cpu 32 --excel --evalue 1.0e-5 pfam_scan.pl -fasta pep.fa -dir PfamScan/data/35.0 -outfile pfam_out.tbl -e_seq1.0e-5 -e_dom 1.0e-5 -cpu 8 blastp -query pep.fa -db tremble_invertebrates -evalue 1.0e-5 -num_threads 16 -out blastp.tremble.out -max_target_seqs. 1 -outfmt 6 -subject_besthit
